# Hydroxyproline-Rich Glycoproteins as Markers of Temperature Stress in the Leaves of *Brachypodium distachyon*

**DOI:** 10.3390/ijms20102571

**Published:** 2019-05-25

**Authors:** Artur Pinski, Alexander Betekhtin, Katarzyna Sala, Kamila Godel-Jedrychowska, Ewa Kurczynska, Robert Hasterok

**Affiliations:** 1 Department of Plant Anatomy and Cytology, Faculty of Biology and Environmental Protection, University of Silesia in Katowice, 40-032 Katowice, Poland; apinski@us.edu.pl (A.P.); robert.hasterok@us.edu.pl (R.H.); 2 Department of Cell Biology, Faculty of Biology and Environmental Protection, University of Silesia in Katowice, 40-032 Katowice, Poland; katarzyna.sala@us.edu.pl (K.S.); kgodel@us.edu.pl (K.G.-J.)

**Keywords:** arabinogalactan proteins, *Brachypodium distachyon*, cell wall, extensins, immunohistochemistry, leaf, RT-qPCR, temperature stress

## Abstract

Plants frequently encounter diverse abiotic stresses, one of which is environmental thermal stress. To cope with these stresses, plants have developed a range of mechanisms, including altering the cell wall architecture, which is facilitated by the arabinogalactan proteins (AGP) and extensins (EXT). In order to characterise the localisation of the epitopes of the AGP and EXT, which are induced by the stress connected with a low (4 °C) or a high (40 °C) temperature, in the leaves of *Brachypodium distachyon*, we performed immunohistochemical analyses using the antibodies that bind to selected AGP (JIM8, JIM13, JIM16, LM2 and MAC207), pectin/AGP (LM6) as well as EXT (JIM11, JIM12 and JIM20). The analyses of the epitopes of the AGP indicated their presence in the phloem and in the inner bundle sheath (JIM8, JIM13, JIM16 and LM2). The JIM16 epitope was less abundant in the leaves from the low or high temperature compared to the control leaves. The LM2 epitope was more abundant in the leaves that had been subjected to the high temperatures. In the case of JIM13 and MAC207, no changes were observed at the different temperatures. The epitopes of the EXT were primarily observed in the mesophyll and xylem cells of the major vascular bundle (JIM11, JIM12 and JIM20) and no correlation was observed between the presence of the epitopes and the temperature stress. We also analysed changes in the level of transcript accumulation of some of the genes encoding EXT, EXT-like receptor kinases and AGP in the response to the temperature stress. In both cases, although we observed the upregulation of the genes encoding AGP in stressed plants, the changes were more pronounced at the high temperature. Similar changes were observed in the expression profiles of the EXT and EXT-like receptor kinase genes. Our findings may be relevant for genetic engineering of plants with increased resistance to the temperature stress.

## 1. Introduction

Plant growth and productivity are compromised by various abiotic stresses, among which are high and low temperature stress. Even short periods of temperature stress may significantly decrease the yield, especially when it occurs during the crucial stages of plant development [1]. As was predicted, heat waves and other extreme temperature events are to become more intense, frequent and long-lasting because of global climate change [2]. Thus, thermal stresses must be better understood in the context of the response and adaptation of plants, which may enable crops with improved thermotolerance to be obtained and bred [3]. 

*Brachypodium distachyon* L. Beauv. (Brachypodium), which is a member of the Pooideae subfamily, is a wild annual grass species that is widespread in the regions of the Mediterranean basin, Western Europe, the Middle East, south-west Asia, north-east Africa, North and South America and Australia. It is closely related to many temperate zone key cereals, including wheat, barley, rye, oats and various forage grasses [4]. Due to its numerous favourable biological features such as a relatively small nuclear genome that ranges from 270 Mb to 350 Mb (depending on the methodology that is used), small stature, self-fertility, a life cycle of less than four months and undemanding growth requirements, *B. distachyon* constitutes an excellent model species [5].

Low-temperature stress results in the downregulation of many photosynthesis-related proteins and, at the same time, the upregulation of the proteins that are involved in reactive oxygen species (ROS) scavenging, redox adjustment, cytoskeletal rearrangements, cryoprotection and cell wall remodelling [6]. Similar results are observed in plants that are stressed by a high temperature [7]. Though the cell wall structure is not primarily altered under heat stress, numerous studies have indicated various changes in its architecture. In low temperature stress, changes in the cell wall rigidity may be an important factor in thermotolerance. Changes in the cell wall are more pronounced in roots because they are more sensitive to temperature stresses than the aerial parts of a plant, though the adverse effect of such stress on leaves directly affects plant productivity. Alterations in the cell wall in response to temperature stress concern cellulose and hemicelluloses biosynthesis, pectin modifications by pectin methylesterases, lignin biosynthesis and changes in the abundance of hydroxyproline-rich glycoproteins (HRGP) [8]. 

HRGP are usually divided into three complex multigene families: (i) arabinogalactan proteins (AGP); (ii) extensins (EXT); and (iii) proline-rich proteins [9]. AGP are further divided into four sub-families according to their polypeptide core: classical AGP, lysine-rich AGP (Lys-rich AGP), arabinogalactan peptides (AG peptides) and fasciclin-like AGP (FLA) [10]. Typically, AGP are strongly O-glycosylated and most of them have glycosylphosphatidylinositol (GPI) anchors that attach the proteins to the plasma membrane, though some of them can be released into the wall matrix *via* GPI cleavage [11]. In connection with their abundance, ubiquitous presence and localisation, AGP play a crucial role in various biological processes such as cell division, cellular communication, programmed cell death, organ abscission, plant-microbe interactions, plant defence and growth as well as in the reproductive processes [12,13,14]. A decrease in the amount of AGP has also been linked with the loss of embryogenic potential in callus cultures of *B. distachyon* [15]. Despite many studies on the role of AGP in plant development, our understanding of their role in the reaction of the plant to temperature stress is still quite limited. Recent studies have shown that temperature stress strongly affects the distribution and content of AGP in the stigma and ovule of *Solanum lycopersicum* as well as in banana leaves and roots, which may indicate that AGP are differentially regulated in the response to temperature stress and that their expression and distribution is tissue specific [16,17,18].

Based on a bioinformatic analysis, EXT were divided into seven classes: classical, short, leucine-rich repeat extensins (LRX), proline-rich extensin-like receptor kinases (PERK), formin-homolog EXT (FH EXT), chimeric and long chimeric EXT. EXT are characterised by the presence of serine, which is followed by three to five proline residues. These prolines are hydroxylated and glycosylated [19]. EXT are known to play important roles in the response to wounding and pathogen infections [20]. This family was also indicated as playing an important role in root-microbe interactions [14,21]. A study on a *B. distachyon* callus showed that one of the chimeric EXT could be considered to be a good marker for embryogenic cells [15]. A chimeric leucine-rich repeat/extensin, LRX1, was shown to be required for root hair morphogenesis in *Arabidopsis thaliana* [22]. However, information on the synthesis and location of extensins in response to temperature stress is scarce.

Thus, the aim of this work was to investigate any changes in the distribution of the epitopes of AGP and EXT in *B. distachyon* leaves through an immunostaining analysis. This approach enabled the distribution of these epitopes and the changes in their leaves that had been stressed by a high or low temperature to be determined. We also determined the level of transcript accumulation of selected genes encoding EXT, EXT-like receptor kinases, and FLA in the leaves of *B. distachyon* that had been stressed by a high or low temperature using RT-qPCR.

## 2. Results

### 2.1. Distribution of the Epitopes of AGP and EXT in Leaves in Response to Temperature Stress

**Figure 1:**
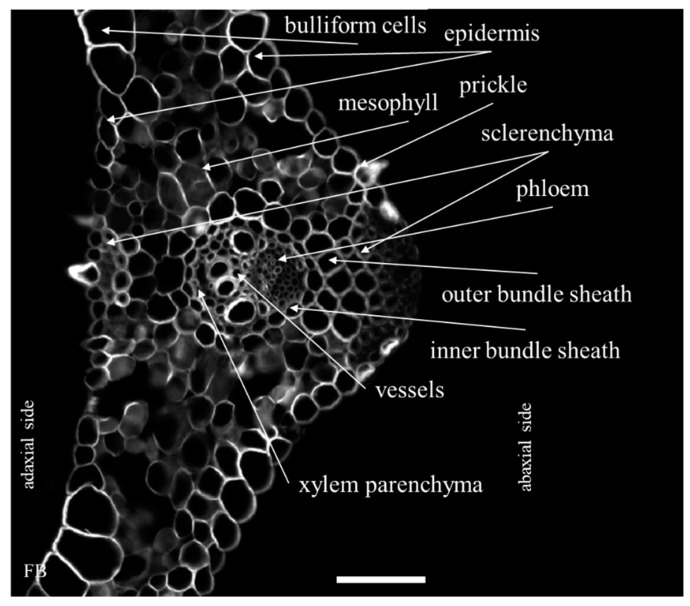
A cross-section of a *B. distachyon* leaf through the major vascular bundle (nomenclature according to Botha [23]) that had been stained with a fluorescent brightener (FB). Scale bar: 50 μm.

**Figure A1:**
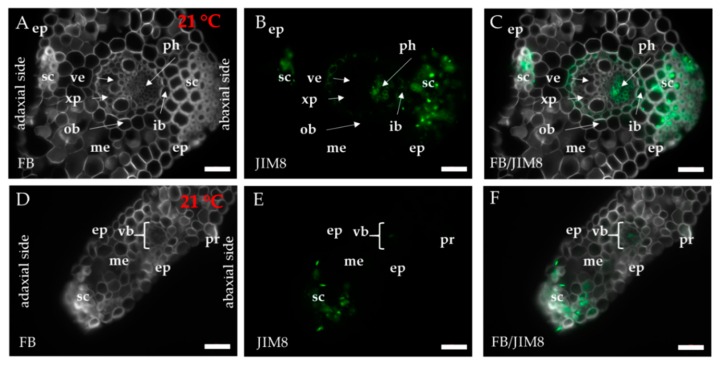
Immunolocalisation of the JIM8 epitope (**A**–**F**) in cross-sections of *B. distachyon* leaves (21 °C), (**A**–**C**): through the major vascular bundle; (**D**–**F**): through the edge of the leaf blade. The white arrows point to the respective parts of the leaf. Abbreviations: ep—epidermis, FB—fluorescent brightener, ib—inner bundle sheath, me—mesophyll, ob—outer bundle sheath, ph—phloem, pr—prickle, sc—sclerenchyma, vb—vascular bundle, ve—vessels, xp—xylem parenchyma. The green colour shows epitope occurrence. Scale bars: 20 μm.

**Figure A2:**
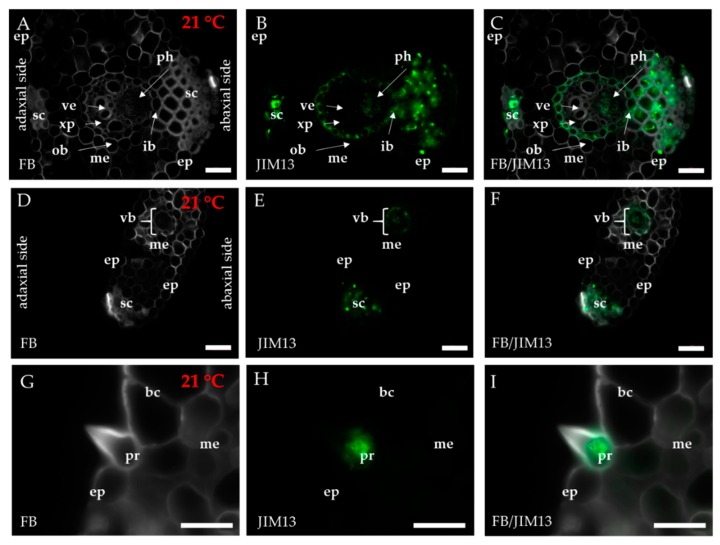
Immunolocalisation of the JIM13 epitope (**A**–**I**) in cross-sections of *B. distachyon* leaves (21 °C), (**A**–**C**): through the major vascular bundle; (**D**–**F**): through the edge of the leaf blade; (**G**–**I**): prickle. The white arrows point to the respective parts of the leaf. Abbreviations: bc—bulliform cells, ep—epidermis, FB—fluorescent brightener, ib—inner bundle sheath, me—mesophyll, ob—outer bundle sheath, ph—phloem, pr—prickle, sc—sclerenchyma, vb—vascular bundle, ve—vessels, xp—xylem parenchyma. The green colour shows epitope occurrence. Scale bars: 20 μm.

**Figure A3:**
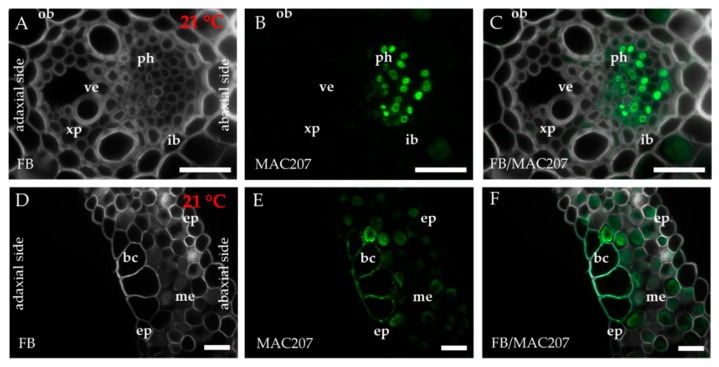
Immunolocalisation of the MAC207 epitope in cross-sections of *B. distachyon* leaves (21 °C), (**A**–**C**): through the major vascular bundle; (**D**–**F**): through the mesophyll and bulliform cells. Abbreviations: ep—epidermis, b—bulliform cells, FB—fluorescent brightener, ib—inner bundle sheath, me—mesophyll, ob—outer bundle sheath, ph—phloem, ve—vessels, xp—xylem parenchyma. The green colour shows epitope occurrence. Scale bars: 20 μm.

**Figure A4:**
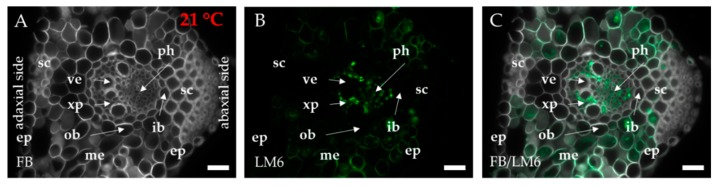
Immunolocalisation of the LM6 epitope (**A**–**C**) in cross-sections of *B. distachyon* leaves (21 °C), through the major vascular bundle. The white arrows point to the respective parts of the leaf. Abbreviations: ep—epidermis, FB—fluorescent brightener, ib—inner bundle sheath, me—mesophyll, o—outer bundle sheath, ph—phloem, sc—sclerenchyma ve—vessels, xp—xylem parenchyma. The green colour shows epitope occurrence. Scale bars: 20 μm.

**Figure A5:**
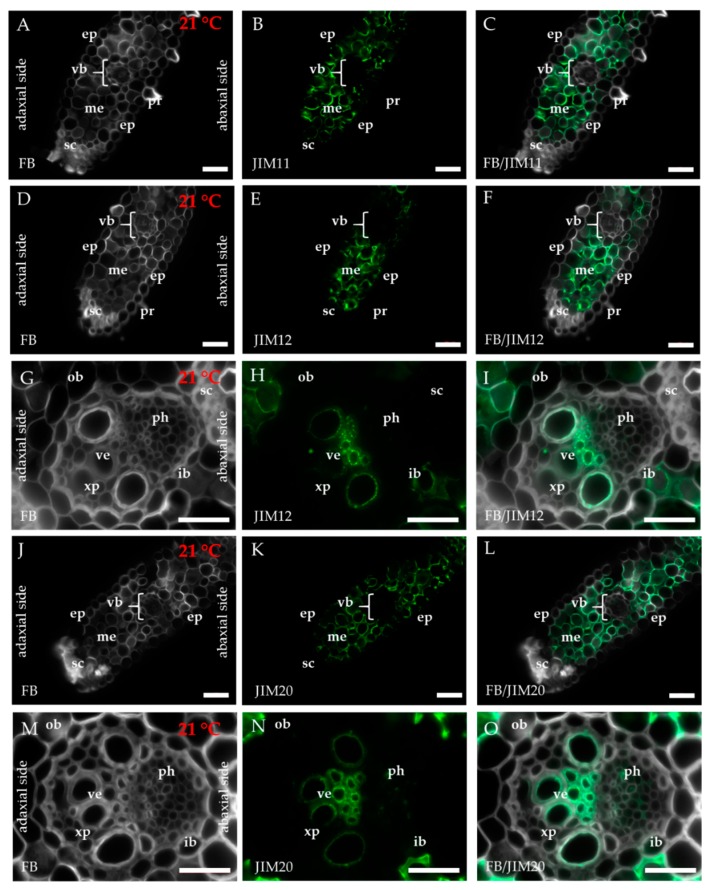
Immunolocalisation of the JIM11 (**A**–**I**), JIM12 (**D**–**I**) and JIM20 (**J**–**O**) epitopes in cross-sections of *B. distachyon* leaves (21 °C), (**A**–**F**,**J**–**L**): through the edge of the leaf blade; (**G**–**I**,**M**–**O**): through the major vascular bundle. Abbreviations: bc—bulliform cells, ep—epidermis, FB—fluorescent brightener, ib—inner bundle sheath, me—mesophyll, ob—outer bundle sheath, p— phloem, pr—prickle, sc—sclerenchyma, vb—vascular bundle, ve—vessels, xp—xylem parenchyma. The green colour shows epitope occurrence. Scale bars: 20 μm.

The distribution of the epitopes of AGP (JIM8, JIM13, JIM16, LM2 and MAC207), pectin/AGP (LM6) and EXT (JIM11, JIM12 and JIM20) in the leaves of *B. distachyon* under normal (21 °C) and thermal stress conditions (4 and 40 °C) was determined. Considering the phenotype, we observed no visible changes induced by the thermal stress. The general anatomy of a *B. distachyon* leaf is shown in Figure 1. In order to present the results clearly, only the antibodies for which changes in their localisation or the intensity of the fluorescence signal in different temperature conditions were observed, are presented in the main text. Figure A1, Figure A2, Figure A3, Figure A4 and Figure A5 show the localisation of the remaining epitopes, in which no changes were identified in their response to temperature treatment.

**Figure 2:**
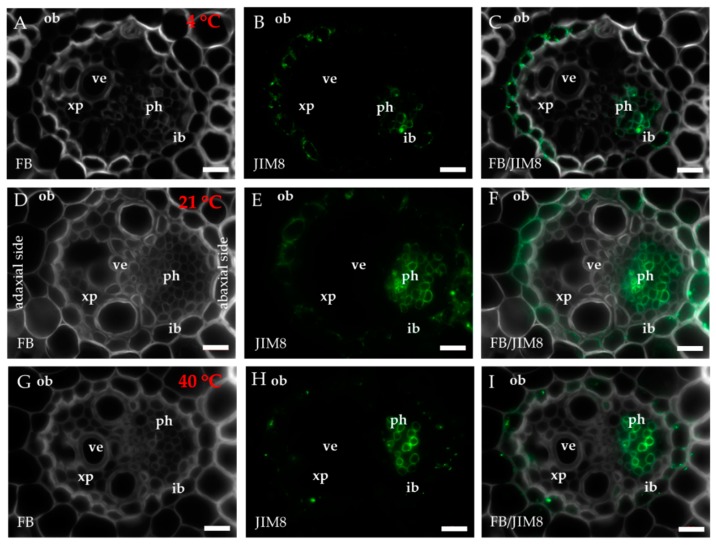
Immunolocalisation of the JIM8 epitope (**A**–**I**) in cross-sections of the *B. distachyon* leaves, (**A**–**I**): through the major vascular bundle. (**A**–**C**): 4 °C; (**D**–**F**): 21 °C; (**G**–**I**): 40 °C. Abbreviations: FB— fluorescent brightener, ib—inner bundle sheath, ob—outer bundle sheath, ph—phloem, ve—vessels, xp—xylem parenchyma. The green colour shows epitope occurrence. Scale bars: 10 μm.

**Figure 3:**
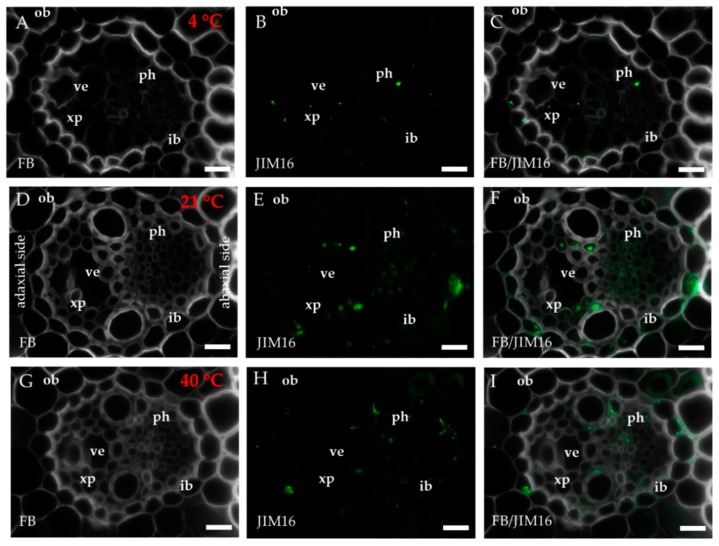
Immunolocalisation of the JIM16 epitope (**A**–**I**) in cross-sections of the *B. distachyon* leaves, (A-I): through the major vascular bundle. (**A**–**C**): 4 °C; (**D**–**F**): 21 °C; (**G**–**I**): 40 °C. Abbreviations: FB—fluorescent brightener, ib—inner bundle sheath, ob—outer bundle sheath, ph—phloem, ve—vessels, xp—xylem parenchyma. The green colour shows epitope occurrence. Scale bars: 10 μm.

**Figure 4:**
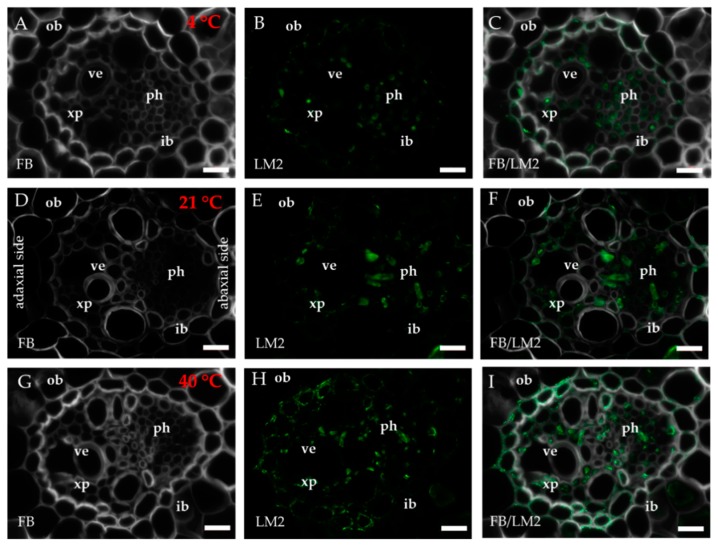
Immunolocalisation of the LM2 epitope (**A**–**I**) in cross-sections of the *B. distachyon* leaves, (**A**–**I**): through the major vascular bundle. (**A**–**C**): 4 °C; (**D**–**F**): 21 °C; (**G**–**I**): 40 °C. Abbreviations: FB—fluorescent brightener, ib—inner bundle sheath, ob—outer bundle sheath, ph—phloem, ve—vessels, xp—xylem parenchyma. The green colour shows epitope occurrence. Scale bars: 10 μm.

**Figure 5:**
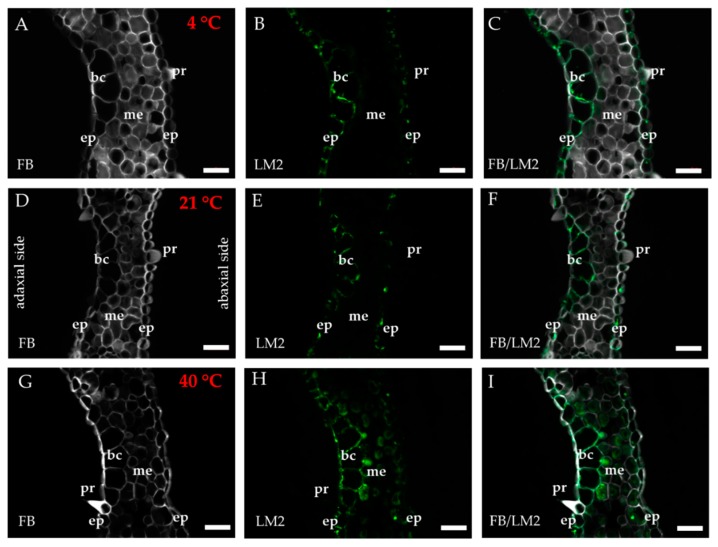
Immunolocalisation of the LM2 epitope (**A**–**I**) in cross-sections of the *B. distachyon* leaves, (**A**–**I**): through the mesophyll and bulliform cells. (**A**–**C**): 4 °C; (**D**–**F**): 21 °C; (**G**–**I**): 40 °C. Abbreviations: bc—bulliform cells, ep—epidermis, FB—fluorescent brightener, me—mesophyll, pr—prickle. The green colour shows epitope occurrence. Scale bars: 20 μm.

**Figure 6:**
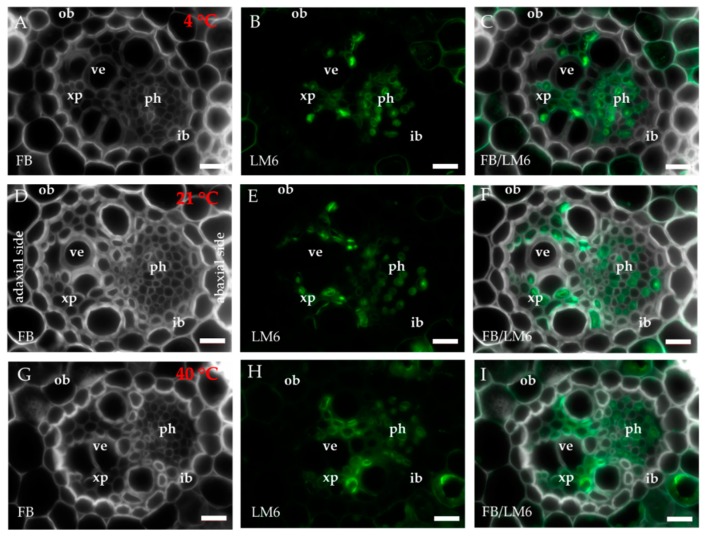
Immunolocalisation of the LM6 epitope (**A**–**I**) in cross-sections of the *B. distachyon* leaves, (**A**–**I**): through the major vascular bundle. (**A**–**C**): 4 °C; (**D**–**F**): 21 °C; (**G**–**I**): 40 °C. Abbreviations: FB—fluorescent brightener, ib—inner bundle sheath, ob—outer bundle sheath, ph—phloem, ve—vessels, xp—xylem parenchyma. The green colour shows epitope occurrence. Scale bars: 10 μm.

The occurrence of the epitopes of AGP was mostly associated with the vascular bundle. The JIM8 epitope was present in the walls and cellular compartments of the inner bundle sheath cells and phloem (Figure 2D–F). This epitope also occurred in the sclerenchyma fibres that were located next to the vascular bundle (Figure A1A–C) or were developing at the edge of the leaf blade (Figure A1D–F). The presence and spatial distribution of the JIM8 epitope were diverse at different temperatures. The JIM8 epitope was less represented in the leaves that were growing at a low temperature (Figure 2A–C). However, in the leaves that had been subjected to a temperature of 40 °C, an increase in the intensity of fluorescence signal was observed in the walls of phloem cells compared to the leaves that were growing at a low temperature (Figure 2G–I). JIM13 was found at the same locations as the JIM8 epitope (Figure A2A–F) and was additionally detected in the intercellular compartments (under intercellular compartments we define the localisation of epitope within the cytoplasm endomembrane system or organelles that are associated with the biosynthesis and secretion pathway to the wall, however these are not visible on the light microscope level [24]) of the prickles (Figure A2G–I). There were no changes in the distribution of the JIM13 epitope or in the intensity of the fluorescence signal between the analysed temperatures. The JIM16 epitope in the control leaves was present in a low amount in the intercellular compartments of the inner and outer bundle sheath cells and phloem as well as in the xylem parenchyma (Figure 3D–F). This epitope was not detected in the xylem parenchyma in the leaves that were growing at 40 °C (Figure 3G–I) and in the leaves from 4 °C, the presence of this epitope was not detected (apart from single dots in the vascular bundle cells; Figure 3A–C). Another AGP epitope, LM2, occurred in the cellular compartments of bundle sheath, phloem and xylem parenchyma in the control leaves (Figure 4D–F). At a low temperature (4 °C), the occurrence of this epitope was very low (Figure 4A–C), while in the leaves that were growing at a high temperature, it was more abundant (Figure 4G–I) compared to the control plants (Figure 4D–F). Additionally, LM2, was detected in the intercellular compartments and/or walls of the epidermis and bulliform cells in the leaves from plants that had been subjected to a high temperature (Figure 5A–I). A signal in the mesophyll cells was detected only in the leaves that were growing at 40 °C (Figure 5G–I). The MAC207 epitope was present in large amounts in the intercellular compartments and/or walls of the phloem cells, mesophyll cells and bulliform cells (Figure A3A–F). At all of the temperatures, its fluorescence had a similar cellular distribution and intensity. The LM6 epitope was detected abundantly in the phloem, xylem parenchyma and, in lower amounts, in the cellular compartments and/or walls of the outer bundle sheath (Figure 6D–F). The fluorescence signal of this epitope in the leaves that were growing at 40 °C was more intense compared to the other temperature treatments (Figure 6G–I vs. Figure 6A–F). Outside the vascular bundle, LM6 was present in the cell walls and/or in the intercellular compartments of the mesophyll cells (Figure A4A–C).

All three extensin epitopes that are recognised by the JIM11, JIM12 and JIM20 antibodies were mostly associated with the mesophyll cells. The JIM11 epitope was present only in the mesophyll cell walls (Figure A5A–C). In addition to occurring in the mesophyll cell walls (Figure A5D–F), JIM12 was also found in the walls of some of the outer bundle sheath cells and vessels (Figure A5G–I). The occurrence of the JIM20 epitope was similar to JIM12 (Figure A5J–O), but had an additional location in the walls and/or cellular compartments of the phloem (Figure A5M–O). All three extensin epitopes occurred abundantly and there were no differences in their distribution or in the intensity of the fluorescence signal among the temperatures that were analysed.

### 2.2. Analysis of the Level of Transcript Accumulation of the Genes Encoding the FLA, EXT and EXT-Like Receptor Kinases

**Figure 7:**
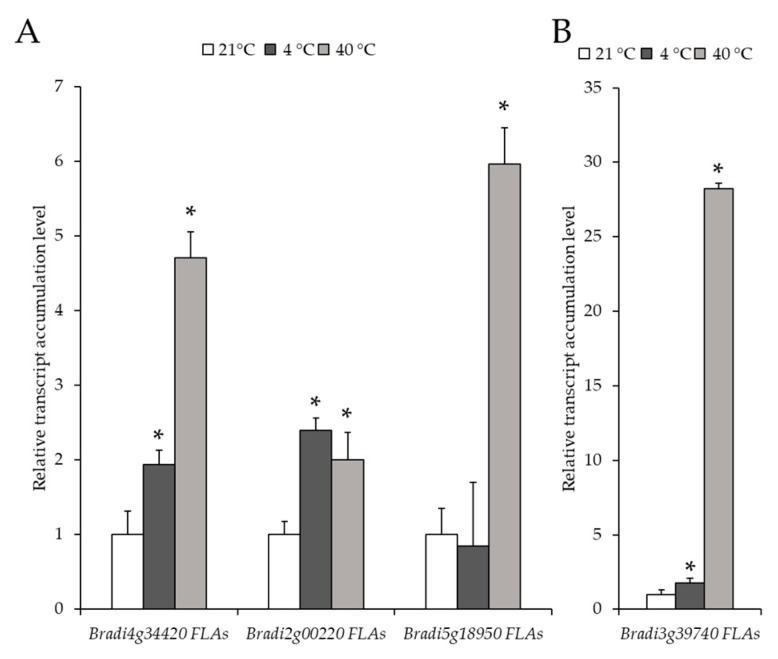
Relative level of transcript accumulation of the fasciclin-like AGP (*FLA)* genes: (**A**) *Bradi4g34420*, *Bradi2g00220* and *Bradi5g18950* and (**B**) *Bradi3g39740*. The relative expression levels were normalised to an internal control (*Bradi1g32860*, gene encoding ubiquitin) and calibrated to the control. Asterisks indicate significant differences from the control using the Student’s t-test (*p* < 0.05; mean ± SD, *n* = 3).

In this study, we determined the level of transcript accumulation of five different genes encoding *FLA* (*Bradi4g34420*, *Bradi2g00220*, *Bradi5g18950*, *Bradi3g39740* and *Bradi2g60270*). The transcript accumulation levels of *Bradi4g34420* and *Bradi2g00220* increased in both temperatures, 4 and 40 °C, compared to the control conditions (Figure 7A). The increase in expression of the *Bradi4g34420* gene at 40 °C (4.7-fold) was higher than at 4 °C (1.9-fold) (Figure 7A). In the case of the *Bradi5g18950* gene, the expression at 4 °C was approximately the same as in the control, while its expression at 40 °C was 6-fold higher (Figure 7A). A similar pattern of expression was found for the *Bradi3g39740* gene, though there was only a slight (1.7-fold) increase in its expression at 4 °C (Figure 7B). Interestingly, there was a dramatic increase (28-fold) in the expression of this gene at 40 °C. Notably, the expression of the *Bradi2g60270* gene was only detectable in the leaves at 40 °C. Generally, temperature stress resulted in a higher level of transcript accumulation of *FLA*, though the increase was more pronounced at 40 °C.

**Figure 8:**
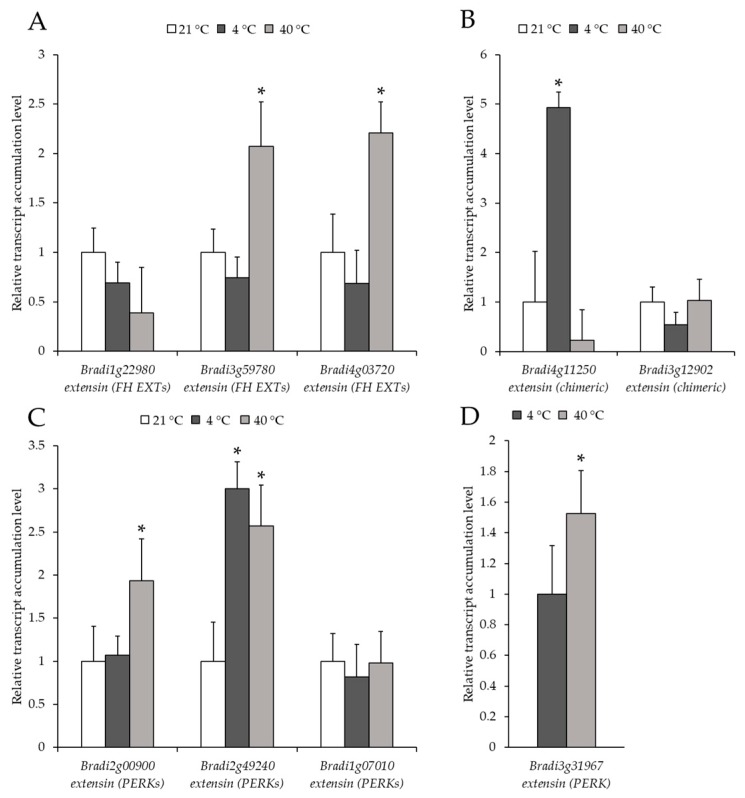
Relative level of transcript accumulation of the extensins (EXT) genes: (**A**) formin-homolog (FH) EXT: *Bradi1g22980*, *Bradi3g59780*, *Bradi4g03720*, (**B**) chimeric EXT: *Bradi4g11250*, *Bradi3g12902*, (**C**) proline-rich extensin-like receptor kinase (PERK): *Bradi2g00900*, *Bradi2g49240*, *Bradi1g07010* and (**D**) *Bradi3g31967*. The relative expression levels were normalised to an internal control (*Bradi1g32860*, gene encoding ubiquitin) and calibrated to the control. Asterisks indicate significant differences from the control using the Student’s t-test (*p* < 0.05; mean ± SD, *n* = 3).

**Figure 9:**
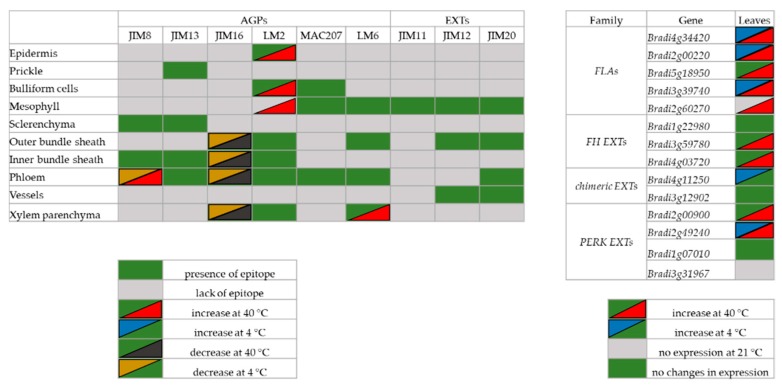
Consolidated results of the distribution of the epitopes EXT and AGP in the leaves of *B. distachyon* and changes in the level of transcript accumulation of the analysed genes.

The level of transcript accumulation of nine different genes encoding EXT and EXT-like receptor kinases were also determined. Each gene was assigned to a group based on its structure: *FH EXT* (*Bradi1g22980*, *Bradi3g59780* and *Bradi4g03720*), *chimeric EXT* (*Bradi4g11250* and *Bradi3g12902*) and *PERK EXT* (*Bradi2g00900*, *Bradi2g49240*, *Bradi1g07010* and *Bradi3g31967*). The level of transcript accumulation of the *Bradi1g22980* gene in the treated plants was not statistically different from the control (Figure 8A). The level of transcript accumulation of two other *FH EXT*, *Bradi3g59780* and *Bradi4g03720* was only statistically higher at 40 °C (Figure 8A). Conversely, the level of transcript accumulation of the *chimeric EXT*, *Bradi4g11250*, increased significantly at 4 °C, though there was no clear difference in its expression for the *Bradi3g12902* gene (Figure 8B). When considering *PERK*, a higher transcript accumulation of the *Bradi2g00900* gene at 4 °C and a higher level of transcript accumulation of the *Bradi2g49240* gene at 4 °C and 40 °C was determined (Figure 8C). Intriguingly, the expression of the other *PERK* gene, *Bradi3g31967*, was only observed in the temperature-stressed samples (Figure 8D). The distribution of all of the epitopes together with changes in the level of transcript accumulation of the analysed genes are summarised in Figure 9.

## 3. Discussion

Although immunohistochemical analyses are widely used to study changes in the chemical components of the cell wall during different developmental processes, in vivo and in vitro information concerning the presence and distribution of the cell wall proteins in leaves that have been subjected to biotic and/or abiotic stresses are scarce and remain largely unexplored [16,18,25]. Previous studies have primarily focused on the differential expression of AGP in response to temperatures stresses in roots and seedlings; however, the involvement of AGP in the response to temperature stress has rarely been studied in leaves [16]. For example, the transient appearance of two AGP proteins in *Triticum aestivum* in response to a low temperature were observed, thus indicating their involvement in the activation of the plant cell defence [16,26]. In transgenic *A. thaliana* plants, non-classical AGP improved the freezing tolerance of seedlings [27]. Temperature stress is one of the factors that limit plant growth and productivity [2,28]. Therefore, data showing changes in the distribution of individual cell wall components, particularly AGP, in connection with temperature stress, are particularly important as the results can be used in genetic engineering for stress tolerance [29].

In the present study, while the distribution of the epitopes of the analysed AGP and EXT was primarily observed in the major vascular bundles (nomenclature according to Botha [23]) and sclerenchyma cells, in the case of the LM2 and MAC207 epitopes, they were present in the mesophyll and epidermal cells, especially in the bulliform cells. Generally, the results for *B. distachyon* presented here are similar to those that have been described for banana leaves in terms of the distribution of epitopes in leaf tissues [18]. In banana, the JIM8 epitope increased in abundance at lower temperatures, thus indicating its role in the tolerance to strong chilling stress [18]. In the pistils of *Solanum lycopersicum* cv Micro-Tom, a high temperature strongly affected the distribution of the JIM8 epitope, which decreased in the stigma and ovule [17]. These varied results with respect to this and other epitopes mean that further intensive studies are necessary. Moreover, such results may indicate that the changes in the chemical composition of the cell walls in response to temperature stress are species-specific. In banana leaves, the presence of the JIM16 epitope was higher in a tolerant cultivar at low temperature stress, thus suggesting that these epitopes may be involved in determining the tolerance of banana to temperature stress [18]. Among the analysed epitopes, the LM2 epitope was detected in most of the leaf tissues and an increase of the LM2 epitope as a response to high temperature stress was observed. A similar distribution was detected in banana leaves, in which this epitope was found in the phloem, bundle sheath, mesophyll and epidermal cells. Low temperature treatment increased the abundance of this epitope in banana [18]. In the leaves of *Tilia x euchlora*, the LM2 epitope was present in the epidermis, hypodermis and parenchyma cells, although as a response to salt stress [25]. Such results may indicate that this wall epitope may be a marker of the plant response to diverse stresses. The abundant presence of the LM6 antibody in *B. distachyon* leaves was found. As LM6 antibody exhibits a high affinity to the (1-5)-α-l-arabinosyl residues, it detects the (1-5)-α-l-arabinan (a pectin rhamnogalacturonan I side chain), however, it can also bind to some AGP (http://www.plantprobes.net/index.php). Thus, the increase in the fluorescence signal in the xylem parenchyma of the temperature-stressed plants may not necessarily indicate changes in the presence of AGP. The function of the arabinan side chains is not well understood and their roles are postulated to be an involvement in the rehydration of the cell wall and flexibility [30,31,32,33]. The more abundant presence of the wall components that are recognised by LM6 antibody, especially in the cytoplasmic compartments, that were observed in our study indicate that leaves react to temperature stress by synthesising and depositing (1-5)-α-l-arabinans into the cell walls.

As has been shown in previous studies, analyses of the distribution and changes in the signal intensity of epitopes can be compared with the expression profiles of the genes encoding the proteins that are targeted by these antibodies [15,34]. In this work, we focused on the FLA that have been implicated in modulation of signalling upstream of cell wall polymer biosynthesis, remodelling, as well as in the stress response as one of the AGP sub-families [35,36,37]. We found an increase in the level of transcript accumulation of the *FLA* in response to temperature stress, which concurs with the immunohistochemistry observations that have been made for the LM2 antibody. It is worth noting that the increase in the level of transcript accumulation of these genes was more pronounced at the high temperature. However, in wheat, four genes encoding *FLA* had a decreased level of transcript accumulation in response to low temperature stress [38]. Similarly, two other *FLA* genes (*OsFLA1* and *OsFLA4*) were downregulated by cold stress in rice [39]. This may reflect intrinsic differences between the analysed species or might be the result of fragmentariness of the conducted experiments, which only focus on a few of the numerous *FLA* genes that are present in a genome. The *Bradi5g18950* gene, which was analysed in our work, exhibited a higher level of transcript accumulation at the high temperature and it was previously shown to be upregulated in a 30-day-old callus that was characterised by an increased embryogenic potential. Conversely, the *Bradi3g39740* gene was linked with a gradual loss of embryogenic potential in *B. distachyon* [15]. Temperature stresses that are induced by low or high temperatures inhibit water uptake, which immediately leads to a slowing down of leaf growth. This observation was correlated with a loss of turgor in leaf cells and an adjustment of the osmoticum, which enables cells to regain turgor [40]. AGP are well known for their water-holding properties [41]. For example, in the resurrection plants, side chains of pectin are highly enriched in arabinose-rich polymers, including AGP. Their presence can prevent water loss during desiccation [8]. In *Coffea arabica* plants that had been subjected to heat stress, there were extensive changes in the cell wall of the leaves. The plants accumulated a higher content of arabinose and galactose, which may suggest that the response of coffee leaves to heat stress is related to type II arabinogalactans and pectins. Moreover, during heat stress, the palisade parenchyma cells were more separated and thinner relative to the control, which resulted in a decreased thickness of the leaves [42]. It has been hypothesised that the organs that are susceptible to water loss such as leaves increase the thickness of the cell walls, thereby limiting desiccation through the production of specific molecules such as AGP [16]. Our results seem to support this hypothesis.

Similar to the temperature stress, salt stress results in a decrease of available water due to a reduction in osmotic potential of the soil solution, which leads to a water deficit [8]. AGP have also been shown to play an important part in the salt stress response and an upregulation of *AGP* in salt-adapted tobacco BY-2 cell cultures was observed. It has been proposed that AGP act as a possible sodium carrier via vesicle trafficking from the apoplast to the vacuoles in salt-adapted tobacco BY-2 cells [43]. A significant upregulation of the *FLA* genes in the salt stress response was observed in the roots of *Populus trichocarpa* [44]. Moreover, AGP were found to act as pectin plasticisers [8,45]. As was shown for an *FLA sos5* (*salt-overly sensitive*) mutant of *A. thaliana*, it exhibits a root-swelling phenotype under salt stress [46]. Further studies showed that the SOS5 protein mediates adherence via its interaction with the cell wall pectin [47]. *At-FLA4* is one of the *FLA* genes in *A. thaliana* that encodes the predicted lipid-anchored glycoprotein and it was shown to positively regulate cell wall biosynthesis and root growth by modulating abscisic acid signalling. Moreover, an *At-fla4* mutant was found to be sensitive to the salt stress [37]. It has been suggested that *At-fla4* might interact with the pectin network via the covalent or non-covalent interactions of its glycans. *At-fla4* may mechanically link pectin with the *AtFei1* and *AtFei2* receptor kinases and the plasma membrane, thus contributing to some biophysical properties such as swelling and interpolymer connectivity [48]. As was indicated by another inactivated mutant of *A. thaliana*, the *FLA1* gene is involved in the early events of lateral root and shoot development in tissue cultures [49]. Considering the salt stress response, it is possible that the upregulation of *FLA* and the increased signal intensity of some of the epitopes of AGP during temperature stress may link with other cell wall polymers such as pectins and thus may modulate the signalling pathways. As has been shown by a number of studies on various species, the pectin content increases during cold stress. Conversely, it decreases during heat stress [8].

An immunohistochemical analysis of the distribution of the *EXT* using the JIM11, JIM12 and JIM20 antibodies showed the presence of all of these epitopes in the mesophyll and the JIM12 and JIM20 epitopes in the vessels. Although no immunohistochemical studies targeting the EXT using the JIM11, JIM12 and JIM20 antibodies were done in the leaves, the distribution of these epitopes has been widely studied in the callus embryos and roots [20,50,51,52,53]. For example, changes in the signal intensity of the JIM11 and JIM12 antibodies were connected with a gradual loss of embryogenic potential in *B. distachyon* callus cultures [15]. Zhang, et al. [54] showed that the EXT that are recognised by the JIM20 antibody were present in the pollen tubes and transmitting tissue of *Nicotiana tabacum* and that the application of hydroxyproline synthesis inhibitor, 3,4-dehydro-L-proline, decreased pollen tube growth. However, studies dedicated to the role of EXT in abiotic stress and especially temperature stress are still scarce. In our study, while we did not observe any changes in the selected epitopes of the EXT in the mesophyll, outer bundle sheath, phloem or vessels at the level of the immunohistochemical analyses, we found an increase of *EXT* and *EXT-like receptor kinase* level of transcript accumulation in the response to temperature stresses, especially during the high temperature stress. This may be partially explained by the fact that we used only three antibodies that bind to the EXT, but it is possible that the application of other anti-EXT antibodies could reveal some changes. Additionally, the effectiveness of immunohistochemical analyses is limited, since it does not provide sufficient resolution and does not focus on individual genes, as is the case of RT-qPCR-based analyses. Changes in the *EXT* gene expression greatly depended on the class of extensins that were being analysed as was shown for *A. thaliana* plants that had been subjected to low temperature stress [55]. Another transcriptomic analysis of the *A. thaliana* response to cold stress showed the downregulation of one of the *PERK* genes [56]. In our experiment, we found that the *PERK extensin*, *Bradi3g31967*, was only expressed in the stressed leaves. The genes that belong to this class have been found in the apical dominance, floral organ defects and root cell elongation [57]. An increased accumulation level of the *PERK4* gene transcript in *A. thaliana* was observed in response to abscisic acid, which is a key regulator of abiotic stress tolerance in plants [58,59]. *PERK1* mRNA from *Brassica napus* was shown to be dramatically and rapidly accumulated in response to wounding and moderately accumulated in response to infection by the fungal pathogen *Sclerotinia sclerotiorum* [60]. Interestingly, the LRX proteins were found to regulate salt tolerance in *A. thaliana*. A triple mutant in the *LRX* genes exhibited a severe salt hypersensitivity and these genes were determined to be an important sensor of the cell wall integrity signals [61]. Moreover, recent studies have hinted at the role of EXT in the plant defence against phytopathogens as well as in interactions with beneficial microorganisms [14,21,62]. EXT have also been implicated in aluminium resistance and its accumulation in the cell walls of pea roots was observed [63]. The changes in the plant cell wall in response to temperature stress are diverse and not only include AGP and EXT, but also alterations in cellulose, hemicellulose, pectin and lignin biosynthesis [8]. Further investigations into the changes in cell wall proteomes could unravel the involvement of other proteins in the stress response because the proteome of the *B. distachyon* cell walls is complex and consists of at least 594 proteins [64,65,66,67,68].

## 4. Materials and Methods

### 4.1. Plant Material

The plants of the *B. distachyon* reference genotype Bd21 that were used is this experiment were cultivated in pots that had been filled with soil mixed with vermiculite at a ratio of 3:1 in a greenhouse. The seeds of *B. distachyon* genotype Bd21 (accession number: PI 254867) were sourced from the collection held by the United States Department of Agriculture—National Plant Germplasm System. The plants were grown in the greenhouse under a 16 h/8 h light/dark photoperiod at 21 ± 1 °C and were illuminated by lamps emitting white light at an intensity of 10 000 lx. For the low temperature stress, the plants were incubated at 4 °C for 24 h and for the high temperature stress, the plants were incubated at 40 °C for 24 h in growing chambers [69]. Plants at the fourth stage of principal growth according to the Hong, et al. [70] were used in this experiment. This stage is referred to as booting and is characterised by the emergence of the head at the top of the growing shoot. The flag leaf was harvested and used to isolate the RNA and to perform the RT-qPCR analysis. For the immunohistochemistry analysis, the middle part of the leaf was collected because this permitted clear observations of the major vascular bundle, epidermis, bulliform cells, mesophyll and sclerenchyma.

### 4.2. Sample Preparation

To determine the chemistry of the cell wall, a set of monoclonal antibodies against the specific cell wall epitopes of the AGP (antibodies JIM8, JIM13, JIM16, LM2, MAC207), pectin/AGP (LM6) and EXT (JIM11, JIM12 and JIM20) (Plant Probes, Leeds, UK) were used. The references and information on the antibodies are shown in Table 1. The leaves were excised, fixed and embedded in Steedman’s wax [20,71]. Transverse sections of the leaf blade (7 μm thick) were cut using a HYRAX M40 rotary microtome (Zeiss, Oberkochen, Germany) and collected on microscopic slides coated with poly-L-lysine (Menzel Gläser, Braunscheig, Germany).

### 4.3. Immunohistochemistry

The sections were de-waxed and rehydrated in an ethanol series (three times in 100, 90 and 50% ethanol in phosphate buffered saline PBS, *v*/*v*, each for 10 min) and PBS (10 min) [71]. The detailed procedure for immunochemical analysis and histological section observation was as previously described [20]. The slides were stained with 0.01% (*w*/*v*) fluorescent brightener 28 (FB) (Sigma-Aldrich, St. Louis, MO, USA) in PBS, which was used to visualise cell walls due to its affinity to cellulose. Two biological replicates were performed with at least eight sections for each replicate.

### 4.4. RT-qPCR

In order to characterise the level of transcript accumulation of the selected genes, RT-qPCR was performed using a LightCycler^®^ 480 SYBR Green I Master in a LightCycler^®^ 480 Real-Time PCR System (Roche, Basel, Switzerland). The total RNA was isolated from the leaves of *B. distachyon*. The primers used in this research are shown in Table A1. The genes encoding extensins with their division into classes were as previously described [19]. The *FLA* genes were selected based on the annotation found in the Phytozome database (https://phytozome.jgi.doe.gov/pz/portal.html). The detailed procedure for RT-qPCR was as in Betekhtin, et al. [72]. Briefly, the isolated RNA were treated with the DNase (QIAGEN, Hilden, Germany), and subsequently used for first-strand cDNA generation. Samples were run in the LightCycler^®^ 480 Real-Time PCR System (Roche, Basel, Switzerland). The PCR conditions were as follow: 5 min at 95 °C, 45 cycles of 10 s at 95 °C, 20 s at 60 °C and 10 s at 72 °C with signal acquisition. Ubiquitin was used as the reference gene and analysis was performed using the 2^−∆∆*C*T^ method. The significant differences between the samples and control were calculated using the Student’s *t*-test.

## 5. Conclusions

In our work, we demonstrated changes in the abundance and diversified expression of the epitopes of the AGP genes encoding FLA, EXT and EXT-like receptor kinases in the leaves of *B. distachyon* in response to temperature stress.

The main findings are as follows:

1. An increase in the JIM8 signal in the walls of phloem cells at 40 °C and a decrease at 4 °C.

2. An increase in the abundance of the LM2 epitope in the leaves of plants that had been subjected to the high temperature.

3. A decrease in the JIM16 signal intensity at 4 and 40 °C.

4. The upregulation of some *FLA*, *EXT* and *EXT-like* receptor *kinases* genes in response to temperature stress (4 and 40 °C).

5. The expression of the *PERK EXT* gene *Bradi3g31967* only in the leaves under low and high temperature stress.

To summarise, our results extend the knowledge about the presence of these epitopes in connection with temperature stress in *B. distachyon*. A precise dissection of the functions of AGP and EXT in response to abiotic stresses and to temperature stress, among others, requires the use of specific mutants whose availability is still limited. However, recent developments in the site-directed mutagenesis techniques such as CRISPR/Cas9 should allow the selective targeting of these genes, which may be helpful to better understand their roles.

## Figures and Tables

**Table 1:** The antibodies that were used for the immunocytochemistry, the epitopes they recognise and the relevant references.

Antibody	Epitope	References
**AGP**		
JIM8	Arabinogalactan	[73]
JIM13	(β)GlcA1->3(α)GalA1->2Rha	[74,75,76]
JIM16	AGP glycan	[74,75,76]
LM2	β-linked GlcA	[75,77]
MAC207	(β)GlcA1->3(α)GalA1->2Rha	[74,75,78,79]
**Pectin/AGP**		
LM6	(1-5)-α-l-arabinosyl residues, can also bind to some AGP	[80,81]
**EXT**		
JIM11	Extensin	[74,82]
JIM12	Extensin	[82]
JIM20	Extensin	[82]

## Abbreviations

AG Peptides	Arabinogalactan peptides
AGP	Arabinogalactan proteins
EXT	Extensins
FH EXT	Formin-homolog EXT
FLA	Fasciclin-like AGP
GPI	Glycosylphosphatidylinositol
HRGP	Hydroxyproline-rich glycoproteins
LRX	Leucine-rich repeat extensins
Lys-rich AGP	Lysine-rich arabinogalactan proteins
PBS	Phosphate-buffered saline
PERK	Proline-rich extensin-like receptor kinases
ROS	Reactive oxygen species
RT-qPCR	Reverse transcription-quantitative polymerase chain reaction

## Appendix A

**Table A1:** The oligonucleotide primers that were used for the RT-qPCR reaction with relevant descriptions of the genes.

Genes	Description of the Genes	Primer Sequence (5′-3′)
*Bradi1g32860*	*ubiquitin*	pF-GAGGGTGGACTCCTTTTGGA pR-TCCACACTCCACTTGGTGCT
***EXT*** **and *EXT*-like receptor kinase**		
*Bradi4g11250*	*extensin (chimeric EXT)*	pF-GCGACTGCGACAATGATGTG
		pR-ACCCCTTGCTAAGCCCTCTA
*Bradi3g12902*	*extensin (chimeric EXT)*	pF-CATCTGGACCTGCCAATGGT
		pR-TCCCAGTTTTGGAGTCTCGC
*Bradi3g59780*	*formin-homolog extensin (FH EXT)*	pF-GATGAATGCCGGAACAGCAC
		pR-GTGGAGAAGAGTGGTGCCTC
*Bradi4g03720*	*formin-homolog extensin (FH EXT)*	pF-GAAGCAGATTGAGGCCGAGA
		pR-CGCGCCTCCATCTTTTGATT
*Bradi1g22980*	*formin-homolog extensin (FH EXT)*	pF-CAGCAGAGCCTGTTGCTTGAC
		pR-TTCTAGGTTTCCGTGCATGAGT
*Bradi2g49240*	*proline-rich extensin-like receptor kinase*	pF-TTCTCAGCCGTTGGGAGATG
	*(PERK)*	pR-GGAAGGTCCCCAAAGTCTCG
*Bradi1g07010*	*proline-rich extensin-like receptor kinase*	pF-CCTCCACGGTAAAGGGCTG
	*(PERK)*	pR-GATCCGTGGATGGCAGTCTT
*Bradi3g31967*	*proline-rich extensin-like receptor kinase*	pF-CCGTCGCCATTAAGAATCTGC
	*(PERK)*	pR-GATTCTTGTGCCGAACTCGC
*Bradi2g00900*	*proline-rich extensin-like receptor kinase* *(PERK)*	pF-TAACTTTGAGGCACAGGTTGCT pR-AGCCATGTATCCAAAAGTCCCC
***FLA***		
*Bradi2g00220*	*fasciclin-like arabinogalactan protein*	pF-AGCTCAACAGCTCCCAGAC pR-CGAAAGCGAGTTGAGCGTG
*Bradi5g18950*	*fasciclin-like arabinogalactan protein*	pF-AATAAAGGGAAGTCACCGTCGC pR-CCGTTCTTCTTGTCATGGACCT
*Bradi4g34420*	*fasciclin-like arabinogalactan protein*	pF-CACATCCTCCAGATGCACGTC pR-CCGGACTCCTGGAACATGG
*Bradi3g39740*	*fasciclin-like arabinogalactan protein*	pF-GTACTATTCCCTGGCGGAGTTC pR-CCATGTTGTCGGTGAGGTTGAG
*Bradi2g60270*	*fasciclin-like arabinogalactan protein*	pF-AGCAGAGCAATCCTCTAGTAGC pR-TGGGTTCTTCTCGCCATTGTTA
